# Surgical Management of Hirschsprung's Disease: A Comparative Study Between Conventional Laparoscopic Surgery, Transumbilical Single-Site Laparoscopic Surgery, and Robotic Surgery

**DOI:** 10.3389/fsurg.2022.924850

**Published:** 2022-07-04

**Authors:** Wei Li, Minghui Lin, Hai Hu, Quanfeng Sun, Cheng Su, Congjun Wang, Yanqiang Li, Yong Li, Jiabo Chen, Yige Luo

**Affiliations:** Department of Pediatric Surgery, The First Affiliated Hospital, Guangxi Medical University, Nanning, China

**Keywords:** hirschsprung's disease, children, conventional laparoscopic surgery, transumbilical single-hole laparoscopic surgery, robotic surgery

## Abstract

**Background:**

Hirschsprung's disease (HD) is a commonly digestive malformation in children that usually requires surgery. This study aims to evaluate the short-term efficacy of conventional laparoscopic surgery (CLS), transumbilical single-hole laparoscopic surgery (TU-LESS), and robotic surgery (RS) in the treatment of Hirschsprung's disease.

**Methods:**

90 patients with Hirschsprung's disease undergone laparoscopic surgery at our center between 2015 and 2019, divided into three groups (group CLS, TU-LESS and RS), were retrospectively analysed.

**Results:**

CLS and TU-LESS group showed no significant difference in operation duration (*P *> 0.05) but shorter operation duration than the RS group (*P *< 0.05). RS group had highest overall SCAR scores, while TU-LESS group had the lowest one (*P *< 0.05). Other parameters such as operative blood loss, hospital stays, recovery time of digestive function, postoperative complications had no significant difference among the three groups (*P *> 0.05).

**Conclusion:**

The three surgical methods for HD revealed similar efficacy, where TU-LESS and CLS spent less time than RS; TU-LESS led to the most aesthetic effect, followed by CLS and RS.

## Introduction

Hirschsprung’s disease (HD) is a common intestinal neuronal developmental disorder that requires surgical removal of the intestinal canal without ganglion cells. In 1995, Georgeson et al. (1) first reported the success of Soave's laparoscopic-assisted endorectal pull-through for HD. With the great advancements in minimally invasive surgery, in 2010, Muensterer et al. reported a single incision laparoscopic-assisted pull-through (SILEP) to treat HD, which includes a single incision in the umbilicus with three channels for operation (2). Since then, some scholars have used this method for the treatment of HD with good therapeutic effects (3, 4, 5). Compared with SILEP, the application of transumbilical laparoscopy single-site surgery (TU-LESS) for the treatment of HD has not yet been reported, which refers to a co-channel in the umbilicus for multiple instruments (6). In 2011, Hebra and colleagues first reported the robot-assisted endorectal pull-through for HD, and in 2020, Pini et al. also reported the robot endorectal pull-through for HD, all of which were successfully performed (7, 8). There has been plenty of studies addressed on the effectiveness of CLS and RS for the treatment of HD, and other minimally invasive surgeries such as single-incision and multi-approach through the umbilicus were already approved. And we expect the surgical effectiveness of TU-LESS that matched CLS and RS.

However, there is still no comparative study on CLS, TU-LESS and RS in the treatment of HD. In this study, clinical data of 90 children diagnosed with HD and who underwent CLS, TU-LESS, RS was collected and analyzed.

## Materials and Methods

### Patients

All patients were given coloclysis in the outpatient clinic for 5–7 days till the drainage fluid became clear with no fecal calculus and their diets were changed to liquid diets. Metronidazole or gentamicin was orally taken one day before the operations, and broad-spectrum antibiotics were given intravenously 0.5 h before the operations.

Inclusion criteria: all of the 90 cases were treated by the same surgeon and the histological diagnosis of HD was confirmed, where the surgical procedure was modified Soave-operation. Exclusion criteria: open operations, other transanal operations, secondary megacolon, other malformations that affected the operation or prognosis and patients younger than 3 years at the last follow-up as age has an impact on children's stool discharging function. In addition, we also excluded 2 cases of total colonic aganglionosis, 8 cases of preoperative colostomy, and 7 cases of incomplete follow-up data. The general information of the patients is shown in Table 1. And studies involving human participants were reviewed and approved by The Medical Ethics Committee of First Affiliated Hospital of Guangxi Medical University.

**Table 1:** Patients' demographics.

Patients	CLS (*n* = 30)	TU-LESS (*n* = 32)	RS (*n* = 28)	*P-*value^a^
Male	20	22	18	0.935
Mean Age, months	4.3 ± 1.3	4..1 ± 1.5	4.3 ± 1.4	0.989
Mean weight, kg	7.2 ± 2.2	7.1 ± 2.2	7.6 ± 2.6	0.773
Transitional zone				0.934
Rectal sigmoid colon	24(80%)	25(78.1%)	23(82.1%)	
Descending colon	6(20.0%)	7(21.9%)	5(17.9%)	

*^a^* *Means Analysis of Variance or rank sum test.*

### Surgical Techniques

The anesthesia method was tracheal intubation general anesthesia combined with sacral block. Patients were put in the supine position at the laparoscopic operation stage, and with their feet elevated at the perineal operation stage. And all the surgeries were performed by the same surgeon who had already completed the learning curve.

**Figure 1:**
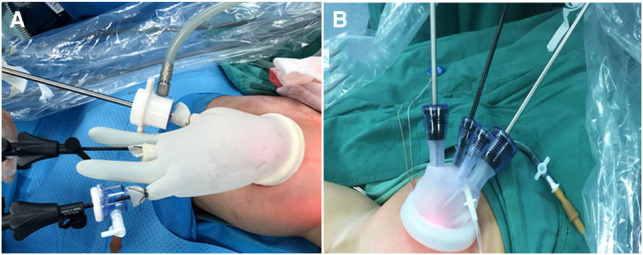
The pictures of homemade glove port (**A**) and multi-instrument laparoscopic ports (**B**) in the TU-LESS, respectively.

**Figure 2:**
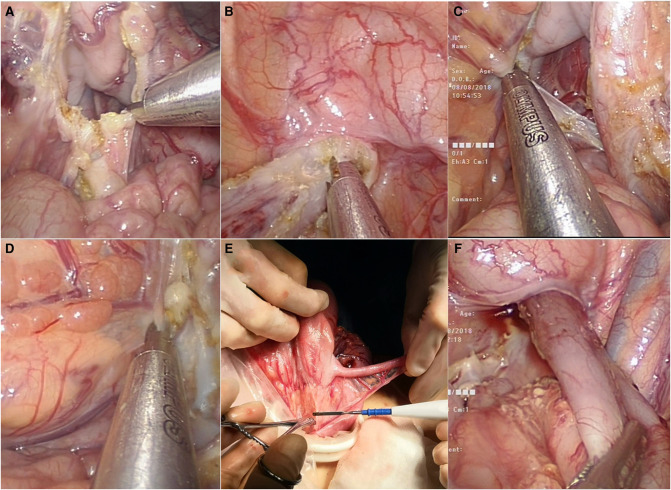
Tu-less. (**A**), cut off sigmoid mesentery and blood vessel; (**B**), free rectum to approximately 3 cm below the peritoneum reflection; (**C**), free splenic flexure of the colon; (**D**), free transverse colon; (**E**), pull the ileocecal bowel out of the abdominal cavity, free ileocecal bowel and remove appendix; (**F**), The ascending colon was inverted and pulled out through the anus, and no Volvulus was confirmed by laparoscopy

Laparoscopic operation stage: though the three surgical procedures are similar, the layouts and methods of the puncture channels are different. For the CLS, a 5 mm channel was impaled in the umbilicus, and at the level of the navel, two 5 mm channels were placed outside the rectus abdominis respectively. For TU-LESS, after the umbilicus was longitudinally incised, the wound retractor was inserted to support a common passage for the instruments in the abdominal wall where a sterile glove (adult single-port laparoscopic puncture device also can be applied in children) was covered, and three 5 mm channels were put through the common passage (see Figure 1). And for RS: a 12 mm channel was set up as a camera port in the 3 cm midline above the umbilicus. An 8 mm channel was set up in the right upper abdomen and the left upper abdomen, respectively, where operating instruments were placed, and a 5 mm channel was set up in the left lower abdomen as auxiliary-hole. Carbon dioxide pneumoperitoneum was established with the pressure of 8–12 mmHg and 2.5–4.5 L/min of gas flow. The scope of the diseased bowel was usually determined by frozen biopsies. An ultrasonic high-frequency cutting and sealing device, though 5 mm channel, was used to cut off the mesentery and lateral peritoneum when the sigmoid colon was lifted, and then dissociated the proximal parts of the colon to 5 cm away from the normal colon, and the distal part was dissociated to 3 cm below the peritoneal reflection (see Figure 2).

Perineal operation stage: the modified Soave endorectal pull-through procedure was applied in all cases. About 0.5–1.0 cm away from the dentate line, the mucosa of the rectum was peeled off, keeping a oblique rectus muscle sheath, where the length of the posterior wall of the rectus muscle sheath was about 1.0 cm and the anterior was about 2.5 cm. The mucosa and muscle of the normal intestine and rectum were stitched up respectively. Before the abdominal puncture incision was sutured, a rubber drainage tube surrounded by Vaseline Gauze was inserted through the anus to facilitate the escape of enteral content. And laparoscopy was performed again to confirm that there was no torsion of the dragged intestine.

After the operation, all patients were given broad-spectrum antibiotics and total parenteral nutrition. Generally, their intestinal function was recovered 1–2 days after the operation (bowel sounds returned (3–5 times/min), drainage of gastric fluid was reduced and clear, and the rectal tube exhausted defecation or gas), when they began to eat a liquid diet. The rubber drainage tube was removed 7 days after the operation, and the anal dilatation treatment was decided 2 weeks after the operation according to the situation of digital rectal examination. The patients were followed up for one month after discharge and every three months for more than one year and then every 3–6 months till they were more than 3 years old.

### Observation Indexes

The observation indexes included sex, age, weight, range of intestinal lesions, operation duration, intraoperative blood loss, hospital stay, recovery time of digestive function, anastomotic leakage, erosions of the perianal region, enterocolitis, adhesive small bowel obstruction, constipation, soiling and the Scar Cosmesis Assessment and Rating (SCAR) scale of patients. Patients were reviewed from the time of surgery to the time of follow-up when they were more than 3 years old when these indexes were evaluated. The definition of recovery of digestive function was bowel sounds returned (3–5 times/min), drainage of gastric fluid was reduced and clear, or the rectal tube exhausted defecation or gas. Anastomotic fistula means a gap of anastomosis in surgical reconstruction of the digestive tract (usually colorectal anastomosis in HD), causing serious infection. Erosions of the perianal region: There are itching, pain and increased secretion in the anus, resulting in skin diseases such as skin erosion, and skin ulcer. The post-operative enterocolitis was defined as the presence of vomiting or explosive diarrhea, abdominal distension, fever and leukocytosis. Characterized by abdominal pain, vomiting, distention, and constipation, adhesive small bowel obstruction was diagnosed non-invasively including a history of abdominal operation or exclusion of other causes of bowel obstruction by imaging. The following indicators of defecation were also assessed at the age of 3 years: constipation and soiling. Constipation means the patient defecates less than 3 times a week with laborious defecates, fecal sclerosis, less quantity of feces, which is manageable by changes in diet or not; and soiling means fecal incontinence manifested as the bowels being emptied in places other than the toilet and soil children’s underwear twice per week or more. We notice the SCAR scale, specifically designed for linear scar, which provides a unique combination of an outcome measure designed for linear scars that may be used by examining photographs, rather than live patients, and that may be completed in less than 30 s by most raters (9). And six months after discharge, the scarring of the patients was evaluated and recorded by two doctors in the outpatient department or through photographs of abdominal scars (see Table 2).

**Table 2:** Perioperative data and postoperative follow-up data.

Parameter	CLS (*n* = 30)	TU-LESS (*n* = 32)	RS (*n* = 28)	F /*χ*^2^-value^a^	*P*-value
Operation duration (min)	152 ± 21^b^	162 ± 22^c^	180 ± 21	13.076	<0.001^b,c^
Blood loss (mL)	9.1 ± 2.2	8.9 ± 2.6	10.2 ± 3.2	1.880	0.159
Time to recover digestive function (day)	1.4 ± 0.5	1.5 ± 0.5	1.6 ± 0.6	0.880	0.418
Hospital stays (day)	8.5 ± 0.9	8.8 ± 0.9	8.4 ± 0.6	1.653	0.197
Anastomotic fistula (*n*%)	1(3.3%)	1(3.1%)	1(3.6%)	0.009	0.995
Perianal erosion (*n*%)	8(26.7%)	9(28.1%)	8(28.6%)	0.029	0.986
Enterocolitis (*n*%)	5(16.7%)	6(18.8%)	4(14.3%)	0.214	0.898
Adhesive small bowel obstruction (*n*%)	0	1(3.1%)	1(3.6%)	1.036	0.596
Constipation (*n*%)	1(3.3%)	1(3.1%)	1(3.6%)	0.009	0.995
Soiling (*n*%)	2(6.7%)	1(3.1%)	2(7.1%)	0.565	0.754

*^a^* *Means Analysis of Variance or rank sum test.*

*^b,c^* *Means compare with group RS, P < 0.05.*

### Statistical Analysis

Data was analyzed by the statistical software SPSS 22.0 and a *P* < 0.05 was considered statistically significant. Normally distributed continuous variables were expressed as mean ± standard deviation (x ± SD), and non-normally distributed continuous variables were expressed as the median (the first and third quartiles). Categorical variables were expressed as percentages (%). Data was using Shapiro–Wilk test to evaluate the normality and normal distribution were found in age, weight, operation duration, intraoperative blood loss, hospital stay, recovery time of digestive function (*P* < 0.05), where the T-test was used to analyze the difference of between group dispersion and Analysis Of Variance were used to evaluate the difference between the means of three or more independent groups. *χ*^2^ test was used to compare the sample rates of the two groups and rank-sum test was used to compare the sample rates of the three groups. For the SCAR scale, Mann–Whitney test was used to compare the median of samples between the two groups and Kruskal–Wallis test for the three groups.

## Results

**Figure 3:**
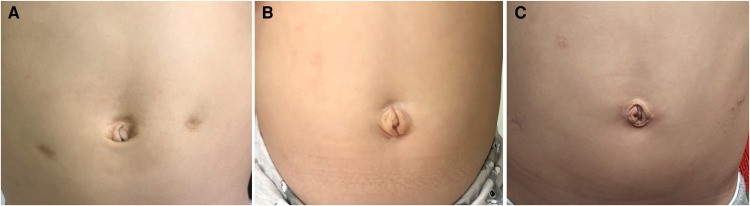
The pictures of the umbilical region of infant undergone CLS (**A**), TUSS-LESS (**B**) and RS (**C**) 6 months after hospital discharge, respectively.

The operations were completed successfully. The operation duration of the CLS group was 152 ± 21 min, TU-LESS group was 162 ± 22 min and 180 ± 21 min for the RS group; there was no statistical difference between the CLS group and TU-LESS group (*P *> 0.05), but the CLS group and TU-LESS group had a shorter operation duration than the RS group (*P *< 0.05). Other indexes such as surgical blood loss, hospital stay, recovery time of digestive function, anastomotic leakage, erosions of the perianal region, enterocolitis, adhesive small bowel obstruction, constipation, soiling did not significantly differ between the three groups (*P *> 0.05) (see Table 3). And the scars of six months after operation among three groups were compared (see Figure 3). The overall SCAR scores of CLS group, TU-LESS group, and RS group were 3, 0 and 4, respectively. TU-LESS group had the lowest scores of scar spread, erythema, hypertrophy/atrophy, overall impression and overall SCAR scores among the three groups (*P *< 0.05). RS group had Significantly higher scores of scar spread, hypertrophy/atrophy, overall impression and overall SCAR scores compared to the CLS group (*P *< 0.05) (see Table 4). In the CLS group, 1 case converted to open operation due to thick and huge intestinal, which could not be pulled out through the anus. 1 case in the TU-LESS group was converted to open operation due to thick and huge intestinal and huge fecal stone, and 1 case was difficult to operate due to thick and huge intestinal and an auxiliary hole was added. In each group, there was 1 case of anastomotic fistula after the operations, which manifested as abdominal pain and fever, and these patients were treated by enterostomy and the stomas were closed 6 months later.

**Table 3:** The Scar Cosmesis Assessment and Rating (SCAR) scale (9).

Parameter	Descriptor	Score
Clinician questions		
Scar spread	None/near invisible	0
	Pencil-thin line	1
	Mild spread, noticeable on close inspection	2
	Moderate spread, obvious scarring	3
	Severe spread	4
Erythema	None	0
	Light pink, some telangiectasias may be present	1
	Red, many telangiectasias may be present	2
	Deep red or purple	3
Dyspigmentation	Absent	0
	Present	1
Suture marks	Absent	0
	Present	1
Hypertrophy/atrophy	None	0
	Mild: palpable, barely visible hypertrophy or atrophy	1
	Moderate: clearly visible hypertrophy or atrophy	2
	Severe: marked hypertrophy or atrophy or keloid formation	3
Overall impression	Desirable scar	0
	Undesirable scar	1
Patient questions		
Itch	No	0
	Yes	1
Pain	No	0
	Yes	1
Total score range	0 (best possible score) to 15 (worst possible score)	

**Table 4:** Cosmetic effect of surgical scars between three different approaches (median, first and third quartiles).

Parameter	CLS	TU-LESS	RS	Kruskal–Wallis test (*P*)	CLS vs TU-LESS (*P*)^a^	CLS vs RS (*P*)^a^	TU-LESS vs RS (*P*)^a^
Scar spread	1(0, 1)	0 (0, 0)	2 (1, 2)	0.000	0.000	0.002	<0.001
Erythema	1(0, 1)	0 (0, 0)	1 (1, 1)	0.000	0.000	0.096	<0.001
Dyspigmentation	0 (0, 0)	0 (0, 0)	0 (0, 1)	0.305	–	–	–
Track marks or suture marks	0 (0, 0)	0 (0, 0)	0 (0, 0)	0.107	–	–	–
Hypertrophy / atrophy	0 (0, 1)	0 (0, 0)	1 (1, 1)	0.000	0.000	0.000	<0.001
Overall impression	0 (0, 0)	0 (0, 0)	0.5 (0, 1)	0.000	0.003	0.045	<0.001
Patient questions	0 (0, 0)	0 (0, 0)	0 (0, 0)	1.000	–	–	–
Overall SCAR scores	3 (3, 3)	0 (0,1)	4(4, 4)	0.000	0.000	0.000	<0.001

*^a^* *Means Mann-Whitney test.*

During the follow-up period, 5 cases in the CLS group developed enterocolitis, where 1 case was hospitalized again, and the other 4 cases were treated with anal dilatation or cleaning enema at home. In the TU-LESS group, 6 cases developed enterocolitis, of which 2 cases were hospitalized again, and the other 4 cases were treated with anal dilatation or cleaning enema at home. And in the RS group, 4 cases were treated with anal dilatation or cleaning enema at home. There was 1 case of adhesive small bowel obstruction in the TU-LESS group and 1 case in the RS group (respectively occurred at 26, and 28 days after surgery), which were cured by conservative treatment and almost no symptoms appeared 3 months after treatment. There was one case of constipation in each group, which was improved by anal dilatation, improving diet, and guiding children to develop fixed defecation habits. There were 2 cases of soiling in the CLS group, 1 case in the TU-LESS group and 2 cases in the RS group, all of which occurred in the early stage after the operation. With the passing of time, anal sphincter training, and rehabilitation, the soiling was significantly relieved.

## Discussion

The surgeries in this study were performed by the same surgeon who had already completed the learning curve. As for a new technique, there was a learning curve for each of the procedures. However, in this study, there was almost no learning curve for the CLP, as a large amount of experience was accumulated by using the CLP to treat HD since 2005. Our center has applied TU-LESS to treat HD since 2015 and has accumulated over 50 operations of experience by 2017(the first 15 cases were performed by TU-LESS with an auxiliary hole), which can be performed skillfully. And before the robotic surgery applied to the treatment of hirschsprung's disease, we had performed more than 20 other operations of the digestive system and urinary system assisted by robots. And with accumulated experience in treating HD by laparoscopic surgeries, we can almost perform the operation nicely, with the mean operation duration of 180 ± 21 min (range: 156–218 min), which was close to the operation duration reported (10).

Many scholars have recognized the effectiveness of laparoscopic surgery for HD because of its small trauma, less bleeding, faster recovery compare to open operation and similar surgical effect (11). CLS can be used to complete the treatment of various types of HD with the relatively short learning curve, simple but highly effective. However, 3–4 puncture scars are left on the abdominal wall after surgery and smaller laparoscopic equipment may help solve this problem.

In 2010, Muensterer *et al* proposed SILS, similar to TU-LESS, for the treatment of HD, where the operation duration was 90–220 min, no intraoperative complications occurred, and the postoperative follow-up was satisfactory (2). Two years later, Tang *et al* reported a comparative study on the short-term effect of SILS and CLS in the treatment of HD, where 28 cases were treated with SILS, with an average operation duration of 122 ± 18 min (2). No intraoperative complications occurred, and the short-term therapeutic effect of the two surgical methods was similar. In 2015, Xia et al reported a comparative study on the mid-term follow-up effect of SILS and CLS in the treatment of HD, where 40 cases of HD were treated with SILS, with an average operation duration of 226 ± 4 min and similar results between the two groups (4). TU-LESS and SILS are similar yet diversified, as both of them were developed based on CLS and are widely used in adults. The difficulty of TU-LESS is the apparatuses and the endoscope camera passed through common canal, more likely to colliding and interfering with each other, while SILS holds 3 different channels similar to CLS. Therefore, it is necessary to be familiar with reverse operation skills, which requires a long learning curve and rich experience in endoscopic operation (12, 13, 14). SILS is characterized by a single port with three fascial incisions, fixed puncture point in bellybutton, a sense of dependence in operation, but relatively fixed scope or space of the operation, and the narrow adjustment range of the instrument and light source, while TU-LESS was a sing hole that multiple equipment pass through, which makes surgeons have relatively abundant space to adjust the apparatuses, and the coordination and cooperation of the equipment is relatively easier. More importantly, after inserting the wound retractor, the original umbilical incision can form a hole with a diameter of 2.0–2.5 cm, through which the intestines with good mobility can be put out of the body for operation, and then it can be easily converted to laparoscopic operation (see Figure 2). Partly supporting the conversion between endoscopic surgery and extra-abdominal surgery is a unique advantage of TU-LESS.

About 60 cases of HD were treated in our center every year, including CLS since 2005, TU-LESS since 2015, and RS since 2018. In this study, the average operation duration of the TU-LESS group was 162 ± 22 min, and no intraoperative complications occurred. The postoperative follow-up effect was similar to that of the CLS group and the RS group, and one of the reasons we think is these patients were undergone modified Soave endorectal pull-through procedure and similar laparoscopic operation stage. Our results showed that under the premise of mature surgical technology and rational use of instruments, TU-LESS could achieve the same clinical effect as CLS and RS, having better operation duration than RS. The biggest advantage of TU-LESS is its aesthetical value as the surgical scar is hidden in the belly button and the cosmetic effect is almost optimal after the operation. Children are different from adults in that the abdominal wall of children is thinner, softer, more mobile, and the intestines are free. In some cases where the intestines are not obviously dilated or hypertrophic, the diseased intestines can be dragged out of the umbilical channel after leaving the sigmoid colon downstream of the endoscope, and intestinal dissociation or biopsy can be carried out outside the abdominal cavity, especially the splenic convoluted intestines of the colon, which can greatly save the operation duration in the abdominal cavity for the resection of long-segment HD. Therefore, the operation duration of the TU-LESS group was similar to that of the CLS group.

In 2001, Meininger *et al* first reported the application of robot-assisted fundoplication in children (15). In 2002, Heller and colleagues reported the application of robot-assisted thoracoscopic surgery (16). Since then, there have been some reports on the application of robots in various systems of pediatric surgery (17–19). In 2011, Hebra et al. reported the application of the Da Vinci Robotic system in the treatment of 12 HD cases (7). The average operation duration was 230 min, and no complications occurred during the operation, while the postoperative follow-up effect was good. In 2020, Pini et al. reported the application of the Da Vinci Robotic system in the treatment of 11 HD cases (8). The average operation duration was 420 min, no complications occurred during the operation, and the postoperative follow-up effect was good. The average operation duration of the RS group in our study was 180 ± 21 min, which was better than the above two reports.

The advantage of the Da Vinci surgical system is that it has a 3D magnified field of view, which has better flexibility and a larger range of motion compared to traditional laparoscopic instruments, thus making the operation more flexible and accurate and clear. By showing the mesangial vascular arch and revealing more clearly the important tissue structures of the rectum and ureter, vas deferens, uterus, and vagina, it can also ensure the blood supply of the intestinal tube and effectively avoiding side injuries with almost no bleeding. However, the disadvantage of the Da Vinci surgical system is that it requires a large operating space. If the operating space is too small, instruments are easy to collide. For the long-segment HD, the use of robotic surgery may be quite a challenge because the field of view needs to be frequently changed due to the wild operation range, though this problem can still be overcome by adjusting the puncture hole. Considering that, RS was more suitable for short-segment and common HD, while CLS and TU-LESS were suitable for all types of HD surgery generally in our experience. And 4 relatively large puncture scars left on the abdominal wall, leaving obvious scars after the operation, is the reason why Scar spread of RS was higher. Maybe single-incision robotic surgery will be a solution to the above problems. Besides, the RS requires good cooperation of assistants and instrument nurse, long time for operation preparation and instrument adjustment, and relatively expensive cost compare to CLS and TU-LESS, a current reason to limit its widespread adoption of it. For example, we looked at these cases and found that their median hospitalization costs were 28,094 yuan, 33,672 yuan and 46,259 yuan for CLS, TU-LESS and RS. Despite many uncertainties, the data still tells something. And comparisons of indications and technical characteristics of three surgical methods were attached (in Table 5).

**Table 5:** Comparison of indications and technical characteristics of three surgical methods.

Age	CLS ≥1 months	TU-LESS ≥3 months	RS ≥1 months
Difficulty of laparoscopic operation	Triangle operation platform is available, which conforms to surgeon	The devices are easy to interfere with each other and need a certain learning curve	Flexible mechanical arm make it more precise and stable
For patients with giant fecal stone or large bowel	Difficult to handle, may require open surgery	Easy to handle, open surgery is not necessary	Difficult to handle, may require open surgery
Surgical field	Clear and stable	More susceptible to interference	Clear 3D visual field
Aesthetic effect (cicatrix)	Visible	Hidden in the umbilicus	Obvious

## Conclusion

The three surgical methods for HD revealed similar efficacy. CLS is a simple operation with a short operation duration that leaves visible scarring. RS has a good field of vision and meticulous operation. Yet, the operation duration is long and surgical scars are quite obvious. TU-LESS has the most cosmetic effect as the scar is hidden in the umbilicus after the operation and flexibility in handling complex surgical situations. Consequently, on the premise of having adequate surgical skills and experience, we recommend TU-LESS in the surgical treatment of HD.

## Data Availability

The raw data supporting the conclusions of this article will be made available by the authors, without undue reservation.
