# Supplementary material for: Surgical Management of Hirschsprung's Disease: A Comparative Study Between Conventional Laparoscopic Surgery, Transumbilical Single-Site Laparoscopic Surgery, and Robotic Surgery
Source: Front Surg. 2022 Jul 4;9:924850. doi: 10.3389/fsurg.2022.924850 (PMC9289258; doi:10.3389/fsurg.2022.924850)
Supplement: Supplementary file 1 [file Table_1_v1.docx]

Supplementary Material

# Supplementary Data

Supplementary Material should be uploaded separately on submission. Please include any supplementary data, figures and/or tables. All supplementary files are deposited to FigShare for permanent storage and receive a DOI.

Supplementary material is not typeset so please ensure that all information is clearly presented, the appropriate caption is included in the file and not in the manuscript, and that the style conforms to the rest of the article. To avoid discrepancies between the published article and the supplementary material, please do not add the title, author list, affiliations or correspondence in the supplementary files.

# Supplementary Figures and Tables

**Tables and Figures**

**Table 1.** Patients^，^Demographics

| Patients | CLS (n=30) | TU-LESS (n=32) | RS(n=28) | *P-value* |
| --- | --- | --- | --- | --- |
| Male | 20 | 22 | 18 | 0.935 |
| Mean Age, months | 4.3±1.3 | 4..1±1.5 | 4.3±1.4 | 0.989 |
| Mean weight, kg | 7.2±2.2 | 7.1±2.2 | 7.6±2.6 | 0.773 |
| Transitional zone |  | | | 0.934 |
| Rectal sigmoid colon | 24(80%) | 25(78.1%) | 23(82.1%) |  |
| Descending colon | 6(20.0%) | 7(21.9%) | 5(17.9%) |  |

**Table 2.** Perioperative data and postoperative follow-up data

| Parameter | CLS  (n=30) | TU-LESS  (n=32) | RS  (n=28) | F /χ^２^-value | *p*-value |
| --- | --- | --- | --- | --- | --- |
| Operation duration (min) | 152±21* | 162±22* | 180±21 | 13.076 | <0.001 |
| Blood loss (ml) | 9.1±2.2 | 8.9±2.6 | 10.2±3.2 | 1.880 | 0.159 |
| Time to recover digestive function (day) | 1.4±0.5 | 1.5±0.5 | 1.6±0.6 | 0.880 | 0.418 |
| Hospital stays (day) | 8.5±0.9 | 8.8±0.9 | 8.4±0.6 | 1.653 | 0.197 |
| Anastomotic fistula (n%) | 1(3.3%) | 1(3.1%) | 1(3.6%) | 0.009 | 0.995 |
| Perianal erosion (n%) | 8(26.7%) | 9(28.1%) | 8(28.6%) | 0.029 | 0.986 |
| Enterocolitis (n%) | 5(16.7%) | 6(18.8%) | 4(14.3%) | 0.214 | 0.898 |
| Adhesive small bowel obstruction (n%) | 0 | 1(3.1%) | 1(3.6%) | 1.036 | 0.596 |
| Unable to control defecation | 0 | 0 | 0 | - | - |
| Constipation (n%) | 1(3.3%) | 1(3.1%) | 1(3.6%) | 0.009 | 0.995 |
| Soiling (n%) | 2(6.7%) | 1(3.1%) | 2(7.1%) | 0.565 | 0.754 |

* means compare with group RS, *P* < 0.05

**Table 3**. The Scar Cosmesis Assessment and Rating (SCAR) scale[9]

| Parameter | Descriptor | Score |
| --- | --- | --- |
| Clinician questions | | |
| Scar spread | None/near invisible | 0 |
|  | Pencil-thin line | 1 |
|  | Mild spread, noticeable on close inspection | 2 |
|  | Moderate spread, obvious scarring | 3 |
|  | Severe spread | 4 |
| Erythema | None | 0 |
|  | Light pink, some telangiectasias may be present | 1 |
|  | Red, many telangiectasias may be present | 2 |
|  | Deep red or purple | 3 |
| Dyspigmentation | Absent | 0 |
|  | Present | 1 |
| Suture marks | Absent | 0 |
|  | Present | 1 |
| Hypertrophy/atrophy | None | 0 |
|  | Mild: palpable, barely visible hypertrophy or atrophy | 1 |
|  | Moderate: clearly visible hypertrophy or atrophy | 2 |
|  | Severe: marked hypertrophy or atrophy or keloid formation | 3 |
| Overall impression | Desirable scar | 0 |
|  | Undesirable scar | 1 |
| Patient questions | | |
| Itch | No | 0 |
|  | Yes | 1 |
| Pain | No | 0 |
|  | Yes | 1 |
| Total score range | 0 (best possible score) to 15 (worst possible score) |  |

**Table 4**.Cosmetic effect of surgical scars between three different approaches (median, first and third quartiles）

| Parameter | CLS | TU-LESS | RS | Kruskal-Wallis test (p) | CLS vs TU-LESS (*p*) | CLS vs RS (*p*) | TU-LESS  vs RS (*p*) |
| --- | --- | --- | --- | --- | --- | --- | --- |
| Scar spread | 1(0, 1) | 0 (0, 0) | 2 (1, 2) | 0.000 | 0.000 | 0.002 | 0.000 |
| Erythema | 1(0, 1) | 0 (0, 0) | 1 (1, 1) | 0.000 | 0.000 | 0.096 | 0.000 |
| Dyspigmentation | 0 (0, 0) | 0 (0, 0) | 0 (0, 1) | 0.305 | - | - | - |
| Track marks or suture marks | 0 (0, 0) | 0 (0, 0) | 0 (0, 0) | 0.107 | - | - | - |
| Hypertrophy / atrophy | 0 (0, 1) | 0 (0, 0) | 1 (1, 1) | 0.000 | 0.000 | 0.000 | 0.000 |
| Overall impression | 0 (0, 0) | 0 (0, 0) | 0.5 (0, 1) | 0.000 | 0.003 | 0.045 | 0.000 |
| Patient questions | 0 (0, 0) | 0 (0, 0) | 0 (0, 0) | 1.000 | - | - | - |
| Overall SCAR scores | 3 (3, 3) | 0 (0,1) | 4(4, 4) | 0.000 | 0.000 | 0.000 | 0.000 |

**Figure 1**

**
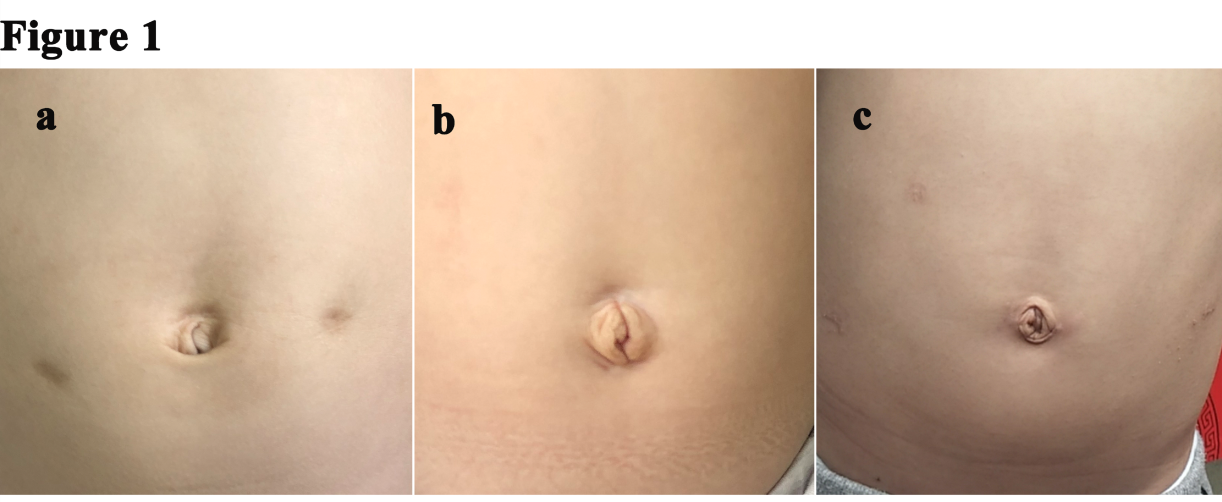
**

The pictures of umbilical region of infant undergone CLS (a), TUSS-LESS (b) and RS (c) 6 months after operation, respectively

**Figure 2**

**
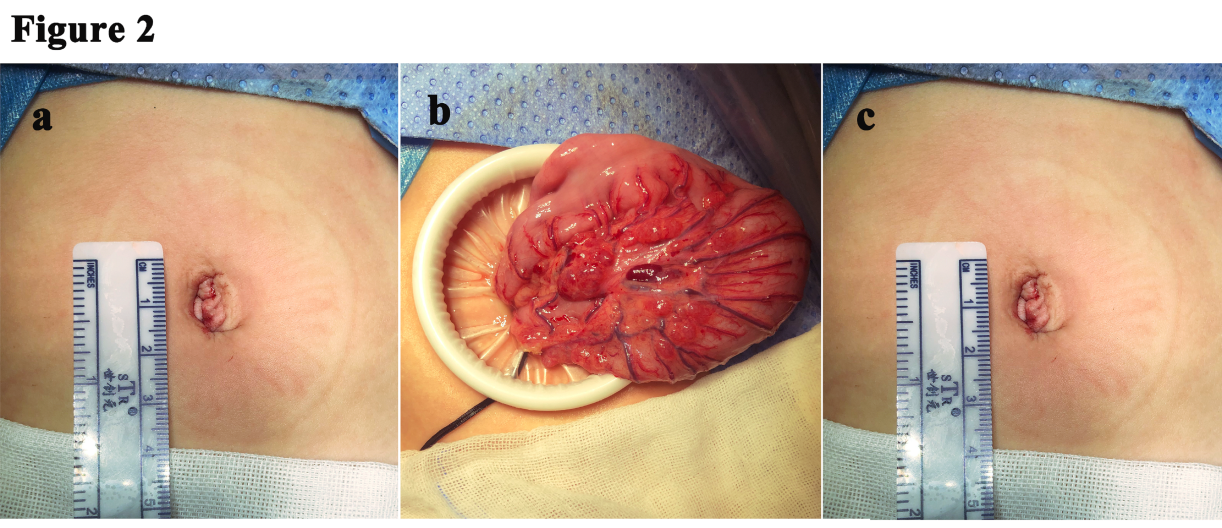
**

The pictures of umbilical region of infant undergone TUSS-LESS at preoperative stage (a), intraoperative stage (b) and postoperative stage (c), respectively
